# A vouchered and georeferenced checklist of the endemic vascular plant species of the Lesser Sunda Islands highlights data deficiencies and threat levels

**DOI:** 10.3897/phytokeys.273.184780

**Published:** 2026-04-09

**Authors:** Laura V. S. Jennings, Liam A. Trethowan, Deby Arifiani, Gemma Bramley, Ruth P. Clark, I. Putu Gede P. Damayanto, Muhammad Faisyal, Carmen Puglisi, Michael Riwu-Kaho, Rosaria Roddie, Andre Schuiteman, Ian Turner, Anna Trias-Blasi, Himmah Rustiami

**Affiliations:** 1 Herbarium, Royal Botanic Gardens Kew, Richmond, Surrey, TW9 3AE, UK Herbarium, Royal Botanic Gardens Kew Richmond United Kingdom; 2 Herbarium Bogoriense, Research Center for Biosystematics and Evolution, The National Research and Innovation Agency (BRIN), Jl. Raya Jakarta-Bogor Km. 46, Cibinong, Bogor, West Java 16911, Indonesia Herbarium Bogoriense, Research Center for Biosystematics and Evolution, The National Research and Innovation Agency (BRIN) Bogor Indonesia; 3 Balai Taman Nasional Gunung Rinjani, Kota Mataram, Nusa Tenggara Barat, Indonesia Herbarium, Missouri Botanical Garden Saint Louis United States of America; 4 Herbarium, Missouri Botanical Garden, 4344 Shaw Blvd., Saint Louis, Missouri 63110-2291, USA University of Nusa Cendana Kupang Indonesia; 5 University of Nusa Cendana, Kupang, Indonesia Oxford SDG Impact Lab, University of Oxford Oxford United Kingdom; 6 Oxford SDG Impact Lab, University of Oxford, Albion House, Littlegate Street, Oxford OX1 1QT, UK Balai Taman Nasional Gunung Rinjani Kota Mataram Indonesia

**Keywords:** Endemic, Indonesia, IUCN Red List, plants, threatened

## Abstract

A checklist of the 319 endemic vascular plants of the Lesser Sunda Islands is presented, derived from specimen data and expert-verified georeferences. The majority of these endemic plant species are single-island endemics (251 species). Timor is the richest in endemic plant species, with 82 single-island endemics, despite Flores (46 endemics) and Sumbawa (38 endemics) being islands of similar size. Of the 121 species that have been Red Listed, 45 are Data Deficient and 56 are in threatened categories. The persistence of many of these species is uncertain, with 51 not collected since the 19^th^ century. We hope that this baseline data will spur targeted searches for these species, highlight taxonomic gaps, and aid conservation planning decisions.

## Introduction

The vascular plants and habitats of the Lesser Sunda Islands (LSI) are under-researched but highly threatened (Miles et al. 2006; Sunderland et al. 2015; Struebig et al. 2022). The region has only partial Flora coverage from the incomplete Flora Malesiana and the Floras of Bali and Lombok (Prawira 1972; Rustiami et al. 2020). The recently published checklists for the whole Sunda–Sahul convergence zone (Joyce et al. 2020) and Indonesia (Sun et al. 2024) are preliminary aggregations of data from multiple sources and are therefore only estimates of the number of endemic species in the LSI. Monk et al. (2012) provide a checklist of LSI endemic flowering plant species and subspecies, which underestimates the number of endemic taxa by at least half and overlaps only partially with this checklist. This checklist of vascular plants complements the recently published checklist for liverworts and hornworts for the same region (Nadhifah et al. 2021).

This checklist will inform conservation prioritisation by highlighting endemic species and their known geographic distributions, allowing targeted searches for species and future protected area planning (Darbyshire et al. 2017; Pusparini et al. 2023). Recent recollections of endemic plant species on Java (Primananda et al. 2022; Primananda et al. 2023) and Sulawesi (Trethowan et al. 2019), not recorded for many decades, indicate that similar searches to recollect LSI endemic species would likely yield results.

The Lesser Sunda archipelago is located within the Wallacea biogeographic zone between the Sunda and Sahul continental shelves. The distinct monsoonal climate has led to the evolution of a unique flora distinct from the rest of the archipelago (Pennington et al. 2009), with physiological adaptations to seasonally dry conditions (Baltzer et al. 2008). Asian seasonally dry forests are under-studied compared to those of the Americas and Africa, with those outside India and Thailand receiving the least research attention (Blackie et al. 2014). The conservation of dry forests is also deprioritised compared to rainforests (Pennington et al. 2018), and there is inadequate protected area coverage for dry forests globally (Miles et al. 2006).

Terminology for the major vegetation types in the LSI differs between publications, which makes analysis of the number of endemics by vegetation type challenging. These forests are often described as savanna in global analyses, which is defined as a fire-adapted forest with an open canopy and an understorey of C4 grasses (e.g. Sunderland et al. 2015); however, this does not adequately capture the local complexity. Savanna fits within the term ‘dry forests’ as defined by CIFOR (Blackie et al. 2015), with ‘seasonally dry forests’ often used in regionally specific accounts (Monk and De Fretes 2012; Hamilton et al. 2020). Six distinct ecoregions are present across the archipelago (Wikramanayake et al. 2002). Monk et al. (2012) define 17 evergreen and deciduous forest subtypes across the archipelago according to total rainfall per annum, length of the dry season, soil type, and elevation, but also state that vegetation classification in this region is ‘unsatisfactory and difficult’. The vegetation of the LSI has not been mapped in detail, and there are local climate, topographic, and geological variations that create a mosaic of different habitats, for example, small areas of ultrabasic rocks in central Timor and limestone in the Tanimbar Islands. This local variability is likely to be important for the conservation of LSI endemic species, but detailed information on the distribution of endemics across ecoregions and extreme soil types is unclear.

The LSI have experienced high levels of forest loss due to conversion to agriculture, with repeated fires leading to conversion to grassland, logging, and settlement development (Monk et al. 2012; Voigt et al. 2021). Due to their seasonal climate, the Lesser Sunda Islands are likely to be vulnerable to changes in rainfall regimes and droughts linked to climate change—induced El Niño events (Allen et al. 2017), particularly as the remaining natural vegetation is highly fragmented. The area has experienced lower than long-term average rainfall since 2005 (Mahrup 2021). Understanding which species are unique to these islands could also inform research into biogeographical and adaptive speciation in this region, as has been demonstrated in the herpetofauna (e.g. Reilly et al. 2022). The lowlands of the LSI are under the greatest pressure from habitat loss; however, the established protected areas in the LSI are mostly in the mountains. In Indonesia, these include those on Gunung Batukaru (Bali), Gunung Rinjani (Lombok), Gunung Tambora (Sumbawa), and Kelimutu (Flores). Timor-Leste has over 30 protected areas, about half of which cover mountains in the central spine of the country.

This paper provides a checklist of endemic species by island for the LSIs, with georeferences provided for all available specimens, which allows us to show the geographical spread of known endemic species by island. These data are a major step in identifying which are the most important areas for endemic and threatened plants across the archipelago (e.g. using Tropical Important Plant Areas criteria).

## Methodology

### Study sites

**Figure 1.**
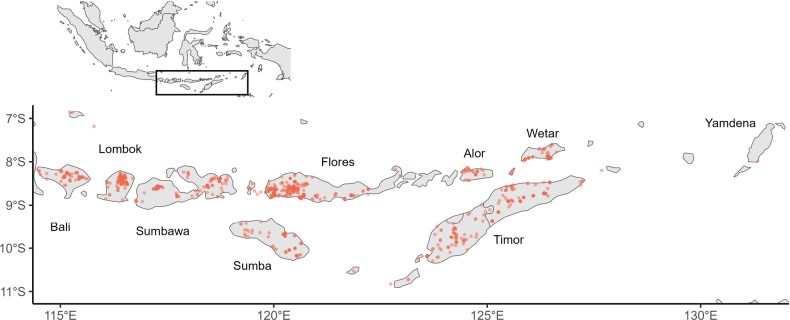
Georeferenced data for 1071 herbarium specimens across the Lesser Sunda Islands.

The study area is defined by the TDWG level 3 area for the Lesser Sunda Islands (TDWG 2001). This consists of the islands between 114°25'E and 132°19'E, the largest of which are Bali, Lombok, Sumba, Sumbawa, Flores, Alor, Wetar, Timor, and Yamdena (Fig. 1). Administratively, these islands are within the countries of Indonesia and Timor-Leste.

### Taxon sampling

A precursor to the rWCVP package (Brown et al. 2023) was used to generate a list of species that only occurred in the Lesser Sunda Islands TDWG level 3 region. This species list was manually refined using herbarium specimen records and by searching the literature for recently published species. We consulted herbaria with major collections from the LSI: A, BO, BM, CANB, G, K, L, P, US, WRSL, and ZT (acronyms follow Thiers) via the Global Biodiversity Information Facility (GBIF) (GBIF.org 2025), in-person visits, and their collection databases. We recorded the threat status for those LSI endemics that have undergone a full IUCN Red List assessment and passed review. We included as endemic those species that occur in the LSI, and any species with occurrences outside the region were removed from the list.

### Georeferencing

All available specimens of the 319 LSI endemic species listed here have been georeferenced (Fig. 1), with an error estimated based on the GBIF best practices, with a minimum of 2 km (as recommended by IUCN). Resources used were Google Earth (Google Earth Pro 2025), GeoNames (GeoNames 2025), and descriptions of collectors’ routes in the Cyclopedia of Malesian Collectors (1974) (van Steenis-Kruseman and van Steenis 1950). The elevation of each specimen was recorded as given on the label or calculated from the georeferenced point, allowing us to show the general pattern of whether the LSI endemics are found more in the lowlands or montane areas. Literature references for the endemic species, which were mainly publications of new species, generic revisions, and Flora Malesiana accounts, were consulted to find further specimens that were misidentified in herbaria and to document those species for which no extant specimens could be found. If these species without known specimens were published with a cited type with a locality description detailed enough to georeference, they were included in the data. Plant form was recorded from herbarium labels, protologues, and, in the rare cases where neither was available, by extrapolation from other members of the genus.

## Results

**Figure 2.**
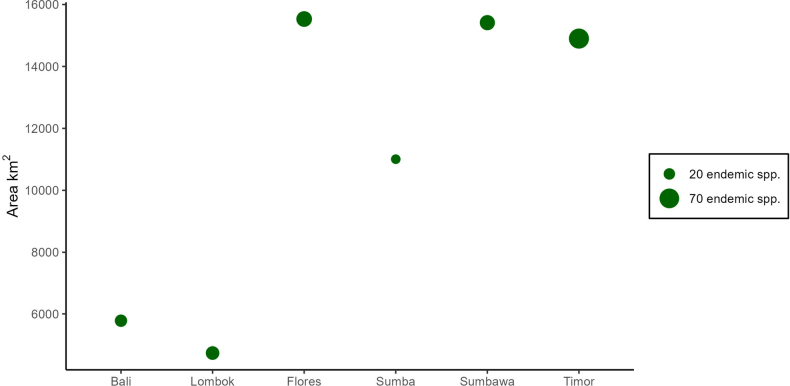
Number of endemic species per island compared to total area.

There are 319 vascular plant species that are endemic to the LSIs. We found 1142 herbarium specimens for 285 of these species; 34 were validly published in the literature, but no voucher specimen could be found. These species are further discussed under ‘uncertain names’ (see Suppl. materials 1–5). There was insufficiently precise geographical information given on the labels to georeference 71 herbarium specimens, leaving us with 1071 georeferenced specimens. There are 68 species endemic to the LSI that are found across multiple islands and 251 single-island endemic species (Fig. 2). Among plant forms, there are 119 woody plants in this checklist, versus 200 herbs, epiphytes, bamboos, and climbers.

**Figure 3.**
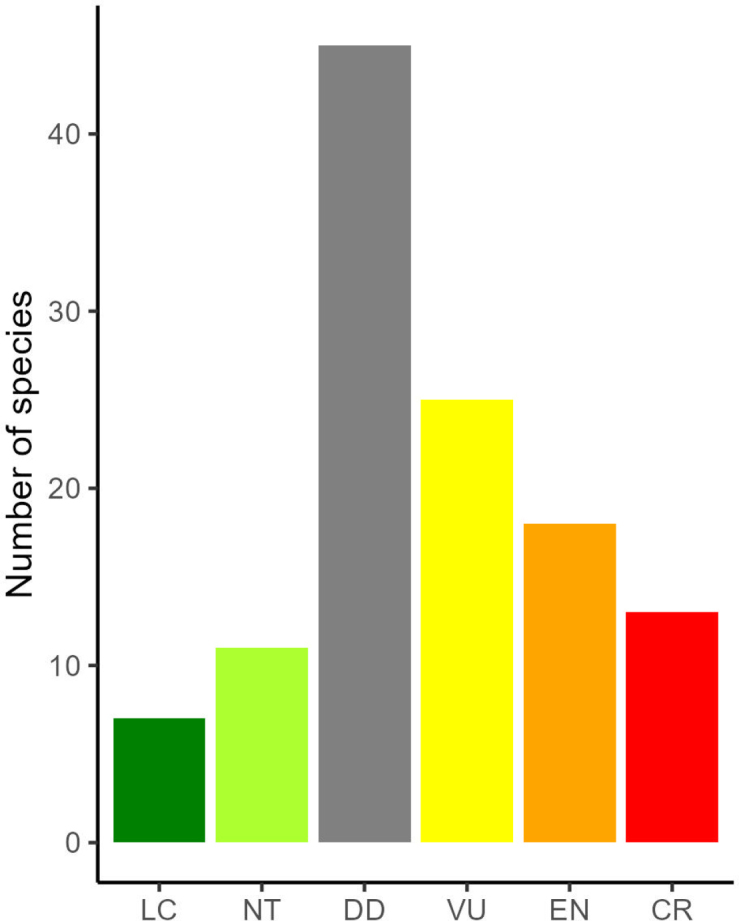
IUCN Red List status for 120 Lesser Sunda Island endemic species that have undergone assessment; 199 Not Evaluated species excluded.

**Figure 4.**
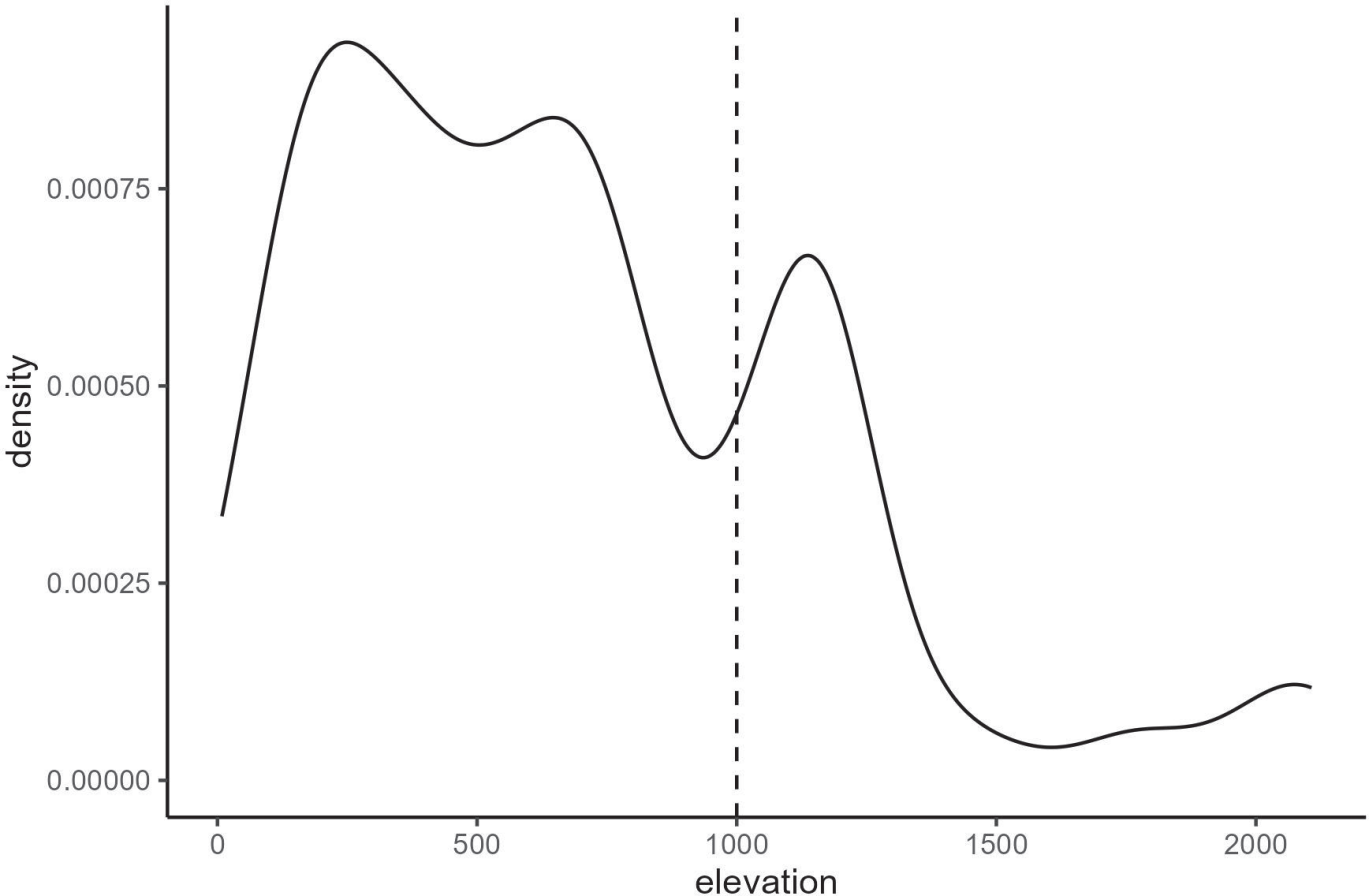
Density plot showing the distribution of 1116 recorded specimens of Lesser Sunda Island endemic species by elevation. The transition from the lowland to montane zone, following van Steenis (1984), is shown at 1000 metres by the dotted line.

The endemic species are found at all elevations, with most occurring below 1000 metres asl (Fig. 4). About two thirds (200 species) of the LSI endemic plants have not been assessed for their extinction risk according to IUCN criteria (IUCN 2001). Of the 119 species that have been assessed, 56 are in the threatened categories Vulnerable, Endangered, and Critically Endangered, 45 are Data Deficient, 12 are Near Threatened, and seven are Least Concern (Fig. 3). The species that have been assessed are across life forms and families. The last known collection dates for the LSI endemics highlight this lack of data. For the 300 species with a known last collection date (19 could not be obtained either through specimens or literature), 51 have not been collected since the 19^th^ century, and only 47 have been collected in the 21^st^ century. We separated IUCN status by island (see Suppl. materials 1–5), but the varying number of species assessed for each island and the large total number of Data Deficient species mean that there are no obvious geographical patterns in IUCN status.

### List of the endemic species of the Lesser Sunda Islands

See Suppl. materials 1–5.

## Discussion

All of the 319 endemic species on this checklist are in need of monitoring, conservation planning, research into their ecological requirements, and, in many cases, taxonomic research and nomenclature corrections. The majority, 251 of the total of 319, are single-island endemics and therefore are likely to have small populations and be vulnerable to stochastic events (Schrader et al. 2024). The 47 species of Orchidaceae are notable as the plant family with the greatest number of endemics, and the only area of the LSI where the orchid flora has been extensively reviewed is Timor (Silveira et al. 2008), so there are undoubtedly more endemic species yet to be described elsewhere in the archipelago. The second- and third-placed plant families are Poaceae and Asteraceae (see Suppl. materials 1–5), likely due to the seasonally dry climate in these islands favouring these species-rich families of open habitats rather than Rubiaceae or Myrtaceae, which are notably diverse in the wetter forests elsewhere in the region.

Many endemic species are known from single specimens or have not been collected since the 19^th^ century. Only about 15% of the endemics have verified sightings in the 21^st^ century. The variation in the number of known endemic species per island is not explained by area. It may be due to differences in surveying effort, although none of the LSI are adequately surveyed for plants (Cowie and Palmerston 2006). Sumba has a particularly small number of endemic species for its size, and this may be due to particularly low collection effort (Sumadijaya et al. 2024). Timor has the highest number of endemic species (82 single-island endemics, and an additional 27 multi-island LSI endemics) and should be considered a priority for plant conservation. There are currently no herbaria in Timor-Leste listed on Index Herbariorum (Thiers), which is likely to make future botanical research into the island’s endemic flora more difficult. Elsewhere in the LSI, there have been few botanical collections from the Tanimbar Islands (the largest of which is Yamdena) other than a recent series of forest plots (Laumonier and Nasi 2018), but these islands have a high percentage of primary vegetation (Margono et al. 2014) and are likely to be as botanically rich as the rest of the LSI, although there is only a single plant species known to be endemic to these islands (*Ochrosia tenimberensis* Markgr. [Apocynaceae]).

The LSI is heavily impacted by human-driven forest loss, particularly in the lowlands, and the most extensive remaining forest areas are in the montane zone (above 1000 metres asl). However, there is no clear relationship between endemism and elevation for LSI endemic species. All the ecosystems of the LSI can be regarded as vulnerable to climate change due to increasingly severe droughts, longer dry seasons, and increased temperatures linked to El Niño events, which interact with fire regimes. Palaeoecological research in Cambodia indicated that Asian dry forests with a mosaic of different forest types are more resilient to a stable shift to savanna than American or African forests (Hamilton et al. 2020), but these limits are unknown in the LSI. However, repeated fires cause a change to grassland dominated by alang-alang [*Imperata cylindrica* (L.) Raeusch.], a vegetation type which has low species diversity and high abundance of other invasives (Usmadi et al. 2020).

The conservation status of the LSI endemic species, where known, fits with the expected pattern of island and endemic species being threatened with extinction (Whittaker and Fernández-Palacios 2007), but the number of species in the Data Deficient category is high at 43% of the total. The threats to the LSI are reasonably well understood, so this is most likely due to a lack of data about the plants themselves. Previous work documenting plant diversity in the region has often focused on woody plants (Whitmore et al. 1989) and forest plots, for example, the extensive recent work in Yamdena (Laumonier and Nasi 2018). However, most endemic species are herbs, epiphytes, or climbers and have not been documented by these methods.

The conservation of these unique endemic species depends on addressing the most urgent basic research gaps: targeted botanical collecting to understand their taxonomy, determine species ranges, and collect data on habitat and ecology. Although there have been a few LSI-focused taxonomic treatments for some groups (e.g. Posthumus 1944; Wijaya 2001), many groups still need revision, notably those most rich in endemic species, Orchidaceae, non-bamboo Poaceae, and Asteraceae.

Timor, with about twice as many known endemic species as any other island, and western Sumbawa, Wetar, and Yamdena, with relatively large areas of primary forest coverage (Margono et al. 2014), could be priority targets for conservation. There are several wild relatives of commercially important crop species that have yet to be recollected, including the Flores coffee, *Coffea floresiana* Borel., which is only known from the type, and the Timor cotton, *Gossypium timorense* Prokh., which has no known extant specimens. The 51 species not collected since the 19^th^ century could be regarded as possibly extinct; however, the lack of targeted searches and low collecting effort on these islands means that they are equally likely to persist. Improved taxonomic knowledge of these species through the methods suggested above will allow more informed study of their evolutionary history and morphological traits. Combining these data will allow us to model future responses to environmental change. We hope that this checklist increases awareness of these species and is used for *in situ* and *ex situ* conservation planning to ensure their survival.

### Notes on uncertain names

#### Johan Baptist Spanoghe’s catalogue of plants of Timor

Prodromus Florae Timorensis (1841) is the cause of some taxonomic confusion, as the written species descriptions are minimal, no specimens are cited, and, although many of Spanoghe’s specimens are in the Naturalis herbarium (L), there are ten endemic species in this publication for which specimens cannot be found. Johan Baptist Spanoghe was a Resident in the Dutch East India Company who collected plants on Timor between 1831 and 1834 and who wrote the manuscript of his Prodromus before his death in Java in 1838. His manuscript was published in Linnaea by von Schlechtendal (Spanoghe 1836). Seventy illustrations were produced to accompany this work but were never published (van Steenis-Kruseman and van Steenis 1950). They are held by Naturalis and are available on Wikimedia Commons, but do not include the species listed below.

Clusiaceae. *Garcinia timorensis* Zipp. ex Span. Linnaea 15: 178 (1841). “Icon 24. Confusae *G. elliptica* DC. et Stalagmites Dulcis. Herb. Timor. p. 112”. Not listed by Lauterbach in his revision of *Garcinia* (Lauterbach 1922). Despite the apparent citation of an illustration, this has not been found.

Convolvulaceae. *Ipomoea reflexa* Span. Linnaea 15: 341 (1841). Spanoghe says “niet verzonden” (not sent). The Flora Malesiana accounts list it as insufficiently known (van Ooststroom and Hoogland 1948).

Elaeocarpaceae. *Elaeocarpus parviflorus* Span. Linnaea 15: 176 (1841). Several homonyms are causing confusion, as all are in use, but no specimen of this species could be located. Non *Elaeocarpus parviflorus* A.Rich. (1984), non *Elaeocarpus parviflorus* Gagnep (1943), non *Elaeocarpus parvifolius* Wall. (nom. nud.).

Orobanchaceae. *Striga spanogheana* Miq. Fl. Ned. Ind. 2: 704 (1857). No Spanoghe collections were found that match this species.

Phyllanthaceae. *Phyllanthus zippelianus* Müll. Arg. A.P.de Candolle, Prodr. 15(2): 433 (1866). Accepted name for nom. illeg. *Phyllanthus cantoniensis* Zipp. ex Span. Linnaea 15: 347 (1841). Non *Phyllanthus cantoniensis* Hornem.

Rubiaceae. *Hymenodictyon timoranum* (Span.) Miq. Fl. Ned. Ind. 2: 153 (1856). Basionym is *Cinchona timorana* Span. Linnaea 15: 315 (1841).

Rubiaceae. *Spermacoce angustifolia* (Span.) Boerl. Handl. Fl. Ned. Ind. 2(1): 144 (1891). Basionym is *Bigelovia angustifolia* Span. Linnaea 15: 320 (1841). Specimens on GBIF under *S. angustifolia* (Span.) Boerl. are from Brazil and should be under *Galianthe angustifolia* (Cham. & Schltdl.) E.L.Cabral. Specimens under *Spermacoce angustifolia* Wall. are from Myanmar and are unlikely to be this species.

Rubiaceae. *Spermacoce pumila* (Span.) Boerl. Handl. Fl. Ned. Ind. 2(1): 144 (1891). Basionym is *Bigelovia pumila* Span. Linnaea 15: 320 (1841). Records on GBIF from Africa and Brazil are *Spermacoce pumila* (DC.) Pohl ex B.D.Jacks.

Rubiaceae. *Spermacoce sociata* (Span.) Boerl. Handl. Fl. Ned. Ind. 2(1): 144 (1891). Basionym is *Bigelovia sociata* Span. Linnaea 15: 320 (1841).

Rutaceae. *Zanthoxylum timoriense* Span. Linnaea 15: 185 (1841). Cited in Hartley’s 1966 review of the genus as “not seen but from description closer to *Toddalia*”. *Toddalia* is now considered a synonym of *Zanthoxylum*.

Urticaceae. *Urtica rubricaulis* Span. The specimen at L under this name [L.1629463] is *Urtica rubricaulis* Hornem. ex Blume, a later homonym, and not this species.

#### Assumed lost at the Berlin (B) herbarium

The types of these species were deposited at B before 1943 and are assumed to have been destroyed (Hiepko 1987). No extant isotypes are known, and no other specimens are known to exist of these species.

Acanthaceae. *Lepidagathis armata*, Lindau Repert. Spec. Nov. Regni Veg. 13: 553 (1915). Type: *Warburg 17114* (B, presumed destroyed). Sumbawa: Keltro Meltro, xi/1888.

Apocynaceae. *Cynanchum sumbawanum*, Warb. Repert. Spec. Nov. Regni Veg. 3: 306 (1907). Type: *Warburg 17201* (B, presumed destroyed). Sumbawa: Bima, date unknown.

Arecaceae. *Calamus sumbawensis* Burret, Notizbl. Bot. Gart. Berlin-Dahlem 15: 802 (1943). Type: *Rensch 649* (B, presumed destroyed). Sumbawa: Batu Dulang, 2/v/1927. In his revision of *Calamus*, Henderson (2020) says that the name is of uncertain application, as the protologue describes a juvenile leaf with numerous, narrow pinnae. Based on the locality, the specimen could have represented *C. melanochaetes* (Blume) Miq., but it is not possible to be sure.

Primulaceae*Ardisia doeringiana* Malm Repert. Spec. Nov. Regni Veg. 41: 296 (1937). Type: Stein 729 (B, presumed destroyed). Timor: Kappan, 16/xi/1932.

Rhamnaceae*Rhamnus sumbawanus* Lauterb. Bot. Jahrb. Syst. 57: 331 (1922). Type: Warburg 17013 (B, presumed destroyed). Sumbawa: Sambori, date unknown.

#### Other problematic species

Apocynaceae. *Dischidia crassifolia* Zipp. ex Schltr. Beibl. Bot. Jahrb. Syst. 92: 8 (1908). Type ‘cultivated at Buitenzorg Botanic Garden’ (no specimen listed). Schlechter (1908) validates a name previously used by Spanoghe, who merely lists the name and says, “not seen”. Whether both uses of the name truly refer to the same plant is unknown.

Begoniaceae. *Begonia timorensis* (Miq.) Golding & Kareg., Phytologia 54: 494 (1984). Basionym *Diploclinium timorense* Miq. Fl. Ned. Ind. 1(1): 692 (1856). Thomas et al. (2023), publishing the third known *Begonia* species for Timor, state that the type material of *B. timorensis* could not be found, although from the description it is likely to be a synonym of the widely distributed *B. longifolia* Blume.

Euphorbiaceae. *Euphorbia sumbawensis* Boiss. A.P.de Candolle, Prodr. 15(2): 26 (1862). Type: Zollinger 1332. Sumbawa. No specimens found. Not cited in Airy Shaw’s checklist of Euphorbiaceae for the region (Airy Shaw 1982).

Malvaceae. *Gossypium timorense* Prokh. Bot. Zhurn. S.S.S.R. 32: 64 (1947). Basionym *G. javanicum* Decne in 1834, Non *G. javanicum* Blume. At the time of description, Decaisne (1834) says that the only specimen in the Musée d’Histoire Naturelle (P) is too incomplete to provide a complete description, but Fryxell (1965), in his revision of *Gossypium*, says that the type has been lost.

Malvaceae. *Grewia viridiflora* Teijsm. & Binn. Natuurk. Tijdschr. Ned.-Indië 27: 39 (1864). “Hab. Ins. Balie Teijsman” is the only specimen detail given in the protologue, with no date or number. No specimens were found matching these details.

Orchidaceae. *Appendicula baliensis* J.J.Sm. Bull. Jard. Bot. Buitenzorg, sér. 3, 9: 152 (1927). Bali. No specimens found. The 1927 protologue says that the species is “described from a living specimen and flowers preserved in alcohol”.

Orchidaceae. *Trichoglottis bimae* Rchb.f. Bonplandia (Hannover) 5: 39 (1857). Sumbawa. Type: Zollinger 1152. This could not be found in the Reichenbach collection at the Zurich herbarium.

Urticaceae. *Pilea riedlei* (Decne.) Blume. Mus. Bot. 2: 55 (1856). Basionym is *Dubrueilia riedlei* Decne. No specimen is listed in the protologue and no specimens found at BR or P where Decaisne’s collections were deposited.

Urticaceae. *Procris ruhlandii* H.Schroet. Repert. Spec. Nov. Regni Veg. 45: 190 (1938). No specimens are referenced in the text, although it is described as being endemic to Bali.

Zingiberaceae. *Costus chrysocephalus* K.Schum. H.G.A.Engler (ed.), Pflanzenr., IV, 46: 410 (1904). The two known specimens at P, both Zippelius s.n. [P01740578; P01740579] were both cultivated at Bogor botanic gardens and no locality is given for the wild source material. The protologue states it was collected by Zippelius in the Lesser Sunda Islands or New Guinea. No collection dates or numbers are given to help determine which of these is the case.

## Additional information

### Artificial Intelligence (AI) use

The authors accept full responsibility for the content of the manuscript, including the disclosure of any use of AI.

No AI tools were used in the preparation of this manuscript.

### Author ORCIDs

L.V.S. Jennings https://orcid.org/0000-0001-6777-2281

L.A. Trethowan https://orcid.org/0000-0002-0326-9828

D. Arifiani https://orcid.org/0000-0002-0381-8663

G. Bramley https://orcid.org/0000-0003-0893-6789

R.P. Clark https://orcid.org/0000-0001-9974-2933

I.P.G.P. Damayanto https://orcid.org/0000-0001-8740-0696

C. Puglisi https://orcid.org/0000-0003-0304-1812

A. Schuiteman https://orcid.org/0000-0001-6701-8158

A. Trias-Blasi https://orcid.org/0000-0001-9745-3222

H. Rustiami https://orcid.org/0000-0002-2260-250X

### Data availability

All of the data that support the findings of this study are available in the main text or Supplementary Information.

## Supplementary materials

Supplementary material 1 List of species endemic to the Lesser Sunda Islands Data type xlsx Explanation note This dataset is a list of the known endemic plant species of the Lesser Sunda islands, with the islands they are known to occur on, IUCN threat status and the last year there is a verified occurrence for each species. https://binary.pensoft.net/file/1589237This dataset is made available under the Open Database License (http://opendatacommons.org/licenses/odbl/1.0/). The Open Database License (ODbL) is a license agreement intended to allow users to freely share, modify, and use this Dataset while maintaining this same freedom for others, provided that the original source and author(s) are credited. Laura V. S. Jennings, Liam A. Trethowan, Deby Arifiani, Gemma Bramley, Ruth P. Clark, I. Putu Gede P. Damayanto, Muhammad Faisyal, Carmen Puglisi, Michael Riwu-Kaho, Rosaria Roddie, Andre Schuiteman, Ian Turner, Anna Trias-Blasi, Himmah Rustiami

Supplementary material 2 IUCN Red List status by island within the Lesser Sunda Islands Data type jpg Explanation note A map of the Lesser Sunda islands with IUCN Red List staus of the species that occur on each island, separated by island. https://binary.pensoft.net/file/1589238This dataset is made available under the Open Database License (http://opendatacommons.org/licenses/odbl/1.0/). The Open Database License (ODbL) is a license agreement intended to allow users to freely share, modify, and use this Dataset while maintaining this same freedom for others, provided that the original source and author(s) are credited. Laura V. S. Jennings, Liam A. Trethowan, Deby Arifiani, Gemma Bramley, Ruth P. Clark, I. Putu Gede P. Damayanto, Muhammad Faisyal, Carmen Puglisi, Michael Riwu-Kaho, Rosaria Roddie, Andre Schuiteman, Ian Turner, Anna Trias-Blasi, Himmah Rustiami

Supplementary material 3 Potential drivers of Red List status: year of last collection, EOO and total number of collections for each species Data type jpg Explanation note Analysis of Red List status against EOO (as an approximation of species range), year of last collection and the total number of collections of each species. Species assessed as CR or DD had older years of last collection, LC species have larger ranges, and there are more collections of LC and NT species. https://binary.pensoft.net/file/1589239This dataset is made available under the Open Database License (http://opendatacommons.org/licenses/odbl/1.0/). The Open Database License (ODbL) is a license agreement intended to allow users to freely share, modify, and use this Dataset while maintaining this same freedom for others, provided that the original source and author(s) are credited. Laura V. S. Jennings, Liam A. Trethowan, Deby Arifiani, Gemma Bramley, Ruth P. Clark, I. Putu Gede P. Damayanto, Muhammad Faisyal, Carmen Puglisi, Michael Riwu-Kaho, Rosaria Roddie, Andre Schuiteman, Ian Turner, Anna Trias-Blasi, Himmah Rustiami

Supplementary material 4 Number of endemic species by family for the Lesser Sunda Islands Data type xlsx Explanation note A summary table with the number of LSI endemic species per plant family. https://binary.pensoft.net/file/1589240This dataset is made available under the Open Database License (http://opendatacommons.org/licenses/odbl/1.0/). The Open Database License (ODbL) is a license agreement intended to allow users to freely share, modify, and use this Dataset while maintaining this same freedom for others, provided that the original source and author(s) are credited. Laura V. S. Jennings, Liam A. Trethowan, Deby Arifiani, Gemma Bramley, Ruth P. Clark, I. Putu Gede P. Damayanto, Muhammad Faisyal, Carmen Puglisi, Michael Riwu-Kaho, Rosaria Roddie, Andre Schuiteman, Ian Turner, Anna Trias-Blasi, Himmah Rustiami

Supplementary material 5 Georeferenced specimen data for Lesser Sunda Islands species Data type xlsx Explanation note This dataset contains occurrence data for Lesser Sunda Islands endemic species, derived from verified specimen records which have been georeferenced. https://binary.pensoft.net/file/1589241This dataset is made available under the Open Database License (http://opendatacommons.org/licenses/odbl/1.0/). The Open Database License (ODbL) is a license agreement intended to allow users to freely share, modify, and use this Dataset while maintaining this same freedom for others, provided that the original source and author(s) are credited. Laura V. S. Jennings, Liam A. Trethowan, Deby Arifiani, Gemma Bramley, Ruth P. Clark, I. Putu Gede P. Damayanto, Muhammad Faisyal, Carmen Puglisi, Michael Riwu-Kaho, Rosaria Roddie, Andre Schuiteman, Ian Turner, Anna Trias-Blasi, Himmah Rustiami
