# Supplementary figures and images for: A vouchered and georeferenced checklist of the endemic vascular plant species of the Lesser Sunda Islands highlights data deficiencies and threat levels
Source: PhytoKeys. 2026 Apr 9;273:21–36. doi: 10.3897/phytokeys.273.184780 (PMC13087668; doi:10.3897/phytokeys.273.184780)

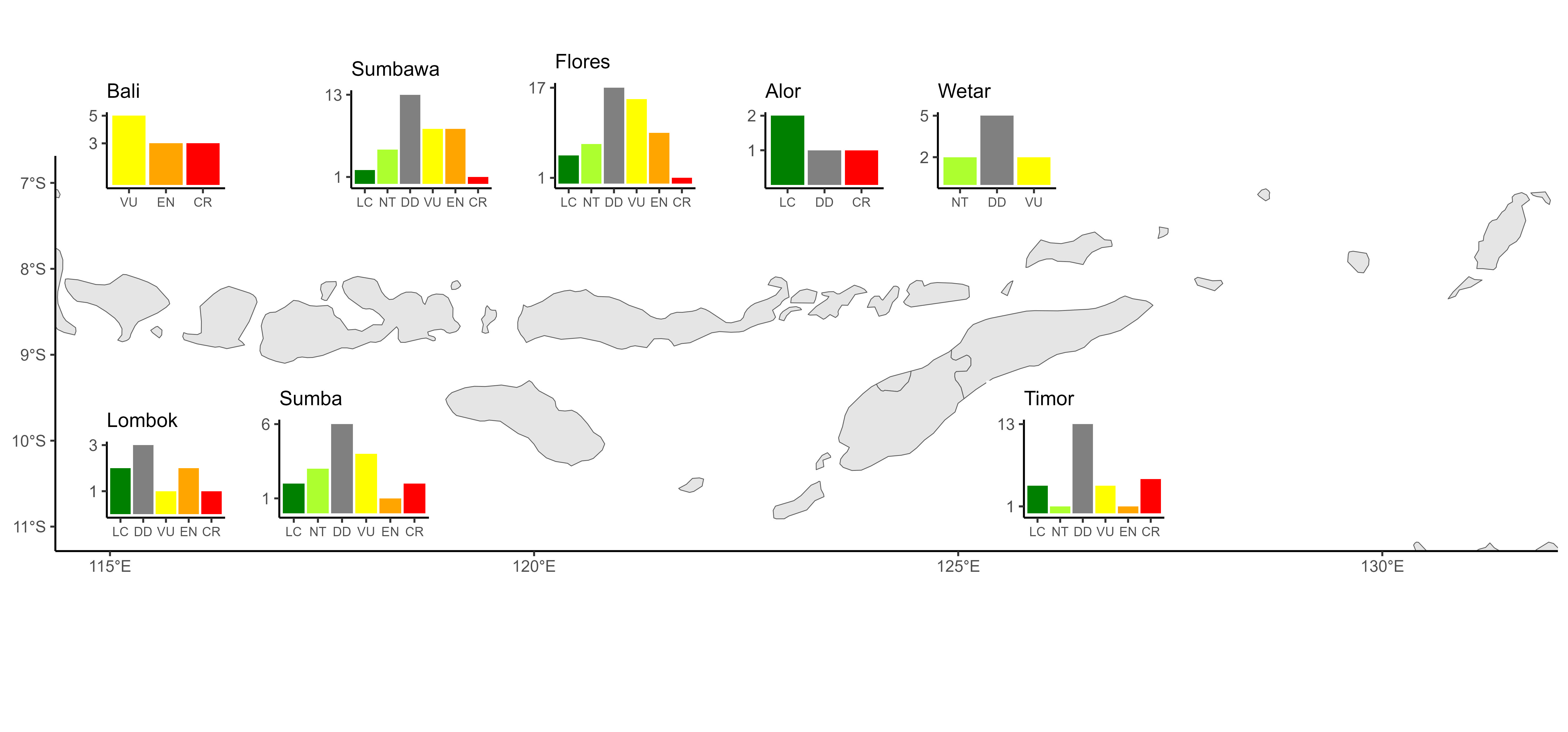

Supplement: Supplementary material 2 — IUCN Red List status by island within the Lesser Sunda Islands [file phytokeys-273-021_article-184780__-s002.jpg]

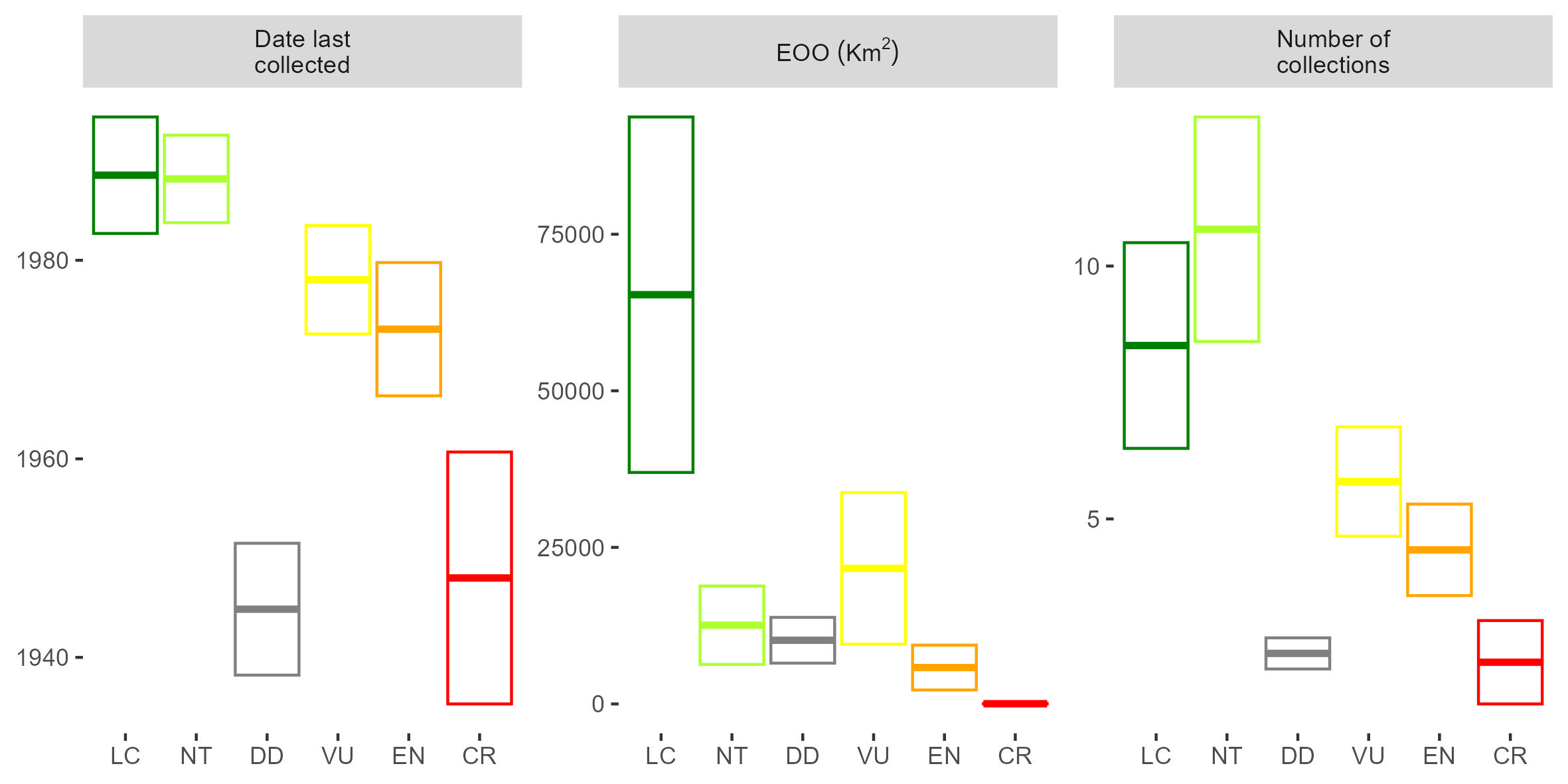

Supplement: Supplementary material 3 — Potential drivers of Red List status: year of last collection, EOO and total number of collections for each species [file phytokeys-273-021_article-184780__-s003.jpg]
